# Antimicrobial Effect of Potassium Iodide Combined with Methylene Blue-Mediated Photodynamic Therapy on Cultured Subgingival Plaque-Derived Polymicrobial Suspensions

**DOI:** 10.3390/pathogens15101031

**Published:** 2026-09-30

**Authors:** Yu Gou, Yuan Chen, Liangjia Bi, Chen He, Shengteng He, Deshu Zhuang

**Affiliations:** 1 Department of Stomatology, The Fourth Affiliated Hospital, Harbin Medical University, Harbin 150001, China; 15145337967@163.com; 2 Department of Stomatology, Sanya Central Hospital Affiliated to Hainan Medical College, Sanya 572022, China; chenyuan_486@163.com (Y.C.);; 3 Division of Periodontology, Hokkaido University, Sapporo 060-0808, Japan; chenhe338@gmail.com

**Keywords:** potassium iodide, methylene blue, photodynamic therapy, plaque-derived polymicrobial suspension, periodontal disease

## Abstract

This study evaluated the antimicrobial efficacy of potassium iodide (KI) combined with methylene blue-mediated photodynamic therapy (MB-PDT) against cultured planktonic polymicrobial suspensions derived from clinical subgingival plaque samples. Bacterial suspensions were assigned to untreated control, light-only, KI-only, MB-PDT, and MB + KI-PDT groups. Neither light irradiation alone nor KI alone significantly affected viable bacterial counts compared with the untreated control. Antibacterial activity was assessed using colony-forming unit (CFU) assays and LIVE/DEAD fluorescence staining, and the virulence-related genes *kgp*, *cdtB*, and *ltxA* were evaluated by RT-qPCR. MB-PDT significantly reduced viable bacterial counts, and the addition of 80 mM KI further enhanced the antibacterial effect. The MB + KI-PDT group showed reductions of approximately 1.89 Log10 CFU/mL compared with the untreated control and 0.45 Log10 CFU/mL compared with MB-PDT alone. LIVE/DEAD staining supported the enhanced antibacterial activity of the combined treatment. The relative transcript levels of *kgp*, *cdtB*, and *ltxA* were also lower after MB + KI-PDT. These findings demonstrate that KI enhanced the antimicrobial activity of MB-PDT in a cultured subgingival plaque-derived planktonic polymicrobial suspension model. Further validation in structured multispecies biofilm models is required.

## 1. Introduction

Periodontitis is an inflammatory disease of the tooth-supporting tissues that can lead to tooth mobility and loss [1]. Persistent colonization by pathogenic microorganisms within the subgingival plaque biofilm contributes to disease initiation and progression [2,3]. The biofilm acts as a reservoir of pathogens and sustains host inflammatory responses, leading to periodontal pocket formation, attachment loss, and alveolar bone resorption [4]. The low-oxygen subgingival environment favors anaerobic and periodontopathogenic bacteria [5]. Periodontal treatment therefore focuses on controlling subgingival plaque. Conventional care relies primarily on scaling and root planing, with adjunctive pharmacological agents when indicated [6]. However, mechanical debridement may not completely remove plaque from deep pockets, furcations, or anatomically complex root surfaces [7], and antimicrobial drugs can cause adverse effects and promote resistance. These limitations support the investigation of additional antimicrobial approaches.

Photodynamic therapy (PDT) is a therapeutic modality based on the interaction among photosensitizing agents, light of specific wavelengths, and molecular oxygen [8]. Upon light activation, photosensitizers undergo photochemical reactions that generate reactive oxygen species, such as singlet oxygen and other oxidative intermediates. These reactive species cause oxidative damage to essential bacterial components, including cell membranes, proteins, and nucleic acids, ultimately resulting in bacterial cell death [9]. Unlike conventional antimicrobial approaches, PDT acts through a multi-target mechanism and demonstrates potent activity against antibiotic-resistant strains. Previous studies have shown that PDT achieves significant antibacterial effects in localized infections, such as wound and burn infections, substantially reducing bacterial load and improving infection management [10].

Methylene blue (MB) is a widely utilized phenothiazine-based photosensitizer in PDT. Upon irradiation with light of specific wavelengths, MB generates reactive oxygen species that induce oxidative damage to bacterial cellular components, including membranes, proteins and nucleic acids, thereby exerting antimicrobial activity [11]. Owing to its favorable water solubility, biocompatibility, and photosensitizing properties, MB-mediated PDT demonstrates potent antibacterial effects, including against multidrug-resistant bacterial strains [12]. Furthermore, this approach exhibits a high safety profile, offering a promising therapeutic strategy for the management of infection-related diseases [13]. Consequently, MB has emerged as a representative photosensitizer in photodynamic therapy research.

Potassium iodide (KI), an inorganic compound characterized by its high aqueous solubility and biocompatibility, has garnered significant interest in medical and oral antibacterial research [14]. Recent investigations have established KI as a potent sensitizer or synergist in PDT. Upon light activation of photosensitizers, KI participates in oxidative processes, yielding iodine radicals, reactive iodine species, and peroxides that exhibit antimicrobial properties, thereby potentiating the eradication of microbial pathogens [15]. Prior research has demonstrated KI’s efficacy in enhancing PDT-mediated inactivation of *Fonsecaea monophora* [16]. Owing to its wide availability, excellent stability, straightforward application, and favorable safety profile, KI presents considerable potential for augmenting the antibacterial efficacy of photodynamic therapy. The pathogenesis of periodontitis reflects complex host-microbe interactions within a dysbiotic subgingival microbial community [17,18].

Although MB-mediated PDT and KI have an established background in antimicrobial research, evidence regarding their activity against cultured polymicrobial suspensions derived from clinical subgingival plaque remains limited. Therefore, the present study evaluated the antimicrobial effect of MB-PDT combined with KI on cultured plaque-derived planktonic microbial suspensions.

## 2. Materials and Methods

### 2.1. Collection of Subgingival Plaque Samples and Preparation of Bacterial Suspensions

Subgingival plaque samples were collected from patients with periodontitis who had not received initial periodontal therapy. Subgingival plaque samples collected from different patients were pooled before bacterial culture. During sampling, subgingival plaque was obtained from periodontal pockets using a scaler. The samples were immediately inoculated onto Columbia blood agar plates and cultured under anaerobic conditions at 37 °C for 4 days. The anaerobic condition was 5% H_2_, 10% CO_2_, and 85% N_2_. After incubation, the colonies were collected and resuspended in sterile saline. The bacterial suspension was then adjusted to an optical density at 600 nm (OD600) of 0.3 and used as the standardized suspension for the following experiments. Because the cultured colonies were harvested and resuspended before treatment, the experimental model represented a cultured subgingival plaque-derived planktonic polymicrobial suspension rather than an intact subgingival biofilm.

### 2.2. Source and Storage of Methylene Blue and Potassium Iodide

Methylene blue used in this study was prepared from a 1% stock solution purchased from Aladdin Reagent Co., Ltd. (Shanghai, China). Potassium iodide was purchased from Xilong Scientific Co., Ltd. (Guangzhou, China). All reagents were stored at 4 °C in the dark.

### 2.3. Treatment

The 660 nm laser system used in this study was supplied by Beijing Honglan Optoelectronic Technology Co., Ltd. (Beijing, China) and had an output power of 200 mW. The laser beam diameter at the sample surface was 15.6 mm, corresponding to an irradiation area of 1.91 cm^2^. The calculated power density was 104.6 mW/cm^2^, and the energy fluence delivered during the 80 s irradiation was 8.37 J/cm^2^. Preliminary concentration screening was performed to determine the working concentrations of MB and KI. MB was tested at final concentrations of 0.0001%, 0.0005%, 0.001%, 0.002%, and 0.004%. KI was tested at final concentrations of 40, 60, 80, 100, and 120 mM in combination with MB-PDT. Based on the preliminary antibacterial results, 0.001% MB and 80 mM KI were selected for subsequent experiments.

The bacterial suspensions were adjusted to an optical density (OD600) of 0.3 before treatment. The suspensions were placed in 24-well plates and positioned 1 cm from the laser device. The irradiation time was 80 s. After dark incubation, the bacterial suspensions were irradiated directly without washing or centrifugation; thus, MB and, where applicable, KI remained in the suspension during irradiation. The pooled bacterial suspension was divided into five groups: an untreated control group, a light-only group, a KI-only group, an MB-PDT group, and an MB + KI-PDT group. The untreated control group received no MB, KI, or laser irradiation. The light-only group was irradiated using the same laser parameters without MB or KI. The KI-only group was incubated with 80 mM KI for 5 min in the dark without laser irradiation. The MB-PDT group was incubated with 0.001% MB for 5 min in the dark and then irradiated with the 660 nm laser for 80 s. The MB + KI-PDT group was incubated with 0.001% MB and 80 mM KI under the same dark conditions and then irradiated using the same laser parameters. Each experiment was performed in triplicate and independently repeated six times (*n* = 6). The mean of the technical replicates from each independent experiment was used for statistical analysis.

### 2.4. Colony-Forming Unit Assay

After treatment, the bacterial suspensions in each group were serially diluted from 10^−1^ to 10^−5^ with sterile saline. Then the diluted suspensions were inoculated onto Columbia blood agar plates and cultured at 37 °C under anaerobic conditions for 1 day. The anaerobic condition was 5% H_2_, 10% CO_2_, and 85% N_2_. After incubation, bacterial viability was evaluated by counting colony-forming units.

### 2.5. Live/Dead Staining and Confocal Observation

After treatment, the LIVE/DEAD BacLight Bacterial Viability Kit (L7012; Invitrogen, Waltham, MA, USA) was added to the bacterial suspensions in each group, and the samples were stained in the dark for 30 min. Then the samples were observed and photographed under a Nikon N2 confocal microscope (Nikon, Tokyo, Japan). Live bacteria stained with SYTO 9 showed green fluorescence, and dead bacteria stained with PI showed red fluorescence. Green and red fluorescence intensities were quantified separately using ImageJ (version 1.54p, National Institutes of Health, Bethesda, MD, USA) and expressed as integrated fluorescence intensity. Identical analysis settings were applied to all images.

### 2.6. RNA Extraction, cDNA Synthesis, and RT-qPCR

After treatment, bacterial suspensions from each group were collected for RNA extraction. Approximately 1 mL of each sample was centrifuged at 4000 r/min, and the bacterial pellet was harvested. Total RNA was extracted using TRIzol reagent combined with mechanical disruption by steel beads. Briefly, the pellet was lysed in 1 mL TRIzol, homogenized using a tissue grinder at 80 Hz for 90 s, and then subjected to chloroform extraction, isopropanol precipitation, and 75% ethanol washing according to the standard TRIzol-based protocol. The RNA pellet was air-dried and dissolved in 10–15 μL DEPC-treated water. RNA concentration and purity were measured using a microspectrophotometer, and RNA samples were stored at −80 °C until further use.

For cDNA synthesis, 500 ng of total RNA was reverse-transcribed in a 20 μL reaction system containing 10 μL 2× ES Reaction Mix, 1 μL Random Primers N9, 1 μL EasyScript RT/RI Enzyme Mix, and RNase-free water. Reverse transcription was performed at 42 °C for 15 min and 85 °C for 5 s, followed by storage at −20 °C.

RT-qPCR was performed using a SYBR-based detection system on an ABI 7300 plus real-time PCR system. Each 20 μL reaction contained 10 μL SYBR qPCR Mix, 0.8 μL of 10 μM forward primer, 0.8 μL of 10 μM reverse primer, 1 μL cDNA template, and 7.4 μL DEPC-treated water; the final concentration of each primer was 0.4 μM. The amplification conditions were as follows: initial denaturation at 95 °C for 5 min, followed by 40 cycles of 95 °C for 10 s, 55 °C for 20 s, and 72 °C for 20 s. Melt-curve analysis produced a single peak for each primer pair, supporting amplification specificity. The target genes were *kgp*, *cdtB*, and *ltxA*. Species-specific normalization was performed: *kgp* was normalized to the *P. gingivalis* 16S rRNA gene, *cdtB* to the *F. nucleatum* 16S rRNA gene, and *ltxA* to the *A. actinomycetemcomitans* 16S rRNA gene. Thus, each target transcript was normalized to the 16S rRNA signal of the corresponding bacterial species rather than to total bacterial 16S rRNA from the mixed suspension. Relative gene expression levels were calculated using the 2^−ΔΔCt method.

The primer sequences used for RT-qPCR are listed in Table 1.

### 2.7. Statistical Analysis

All data were analyzed using GraphPad Prism 10 and are presented as mean ± standard deviation. Differences among multiple groups were analyzed using one-way analysis of variance followed by Tukey’s multiple-comparisons test. A two-sided *p* value < 0.05 was considered statistically significant.

## 3. Results

### 3.1. CFU Assay

**Figure 1:**
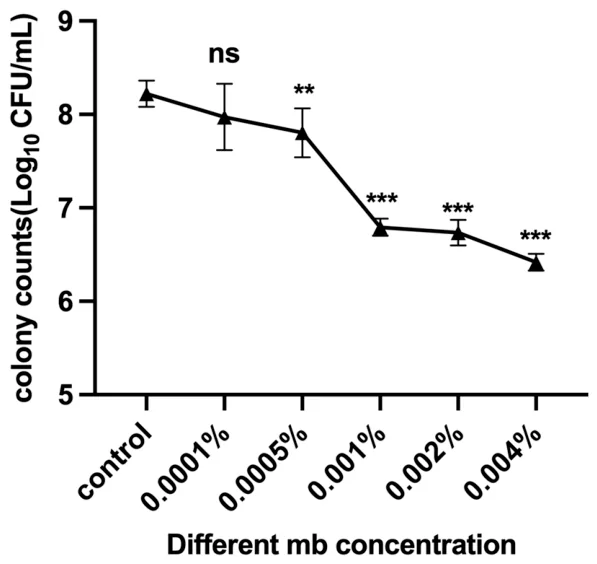
Screening of methylene blue concentrations for MB-PDT. Viable bacterial counts after treatment with the indicated MB concentrations are expressed as Log10 CFU/mL. Data are presented as mean ± SD from six independent experiments (*n* = 6). Statistical comparisons were made with the untreated control group using one-way ANOVA followed by Tukey’s multiple-comparisons test. ns, not significant; ** *p* < 0.01, *** *p* < 0.001.

**Figure 2:**
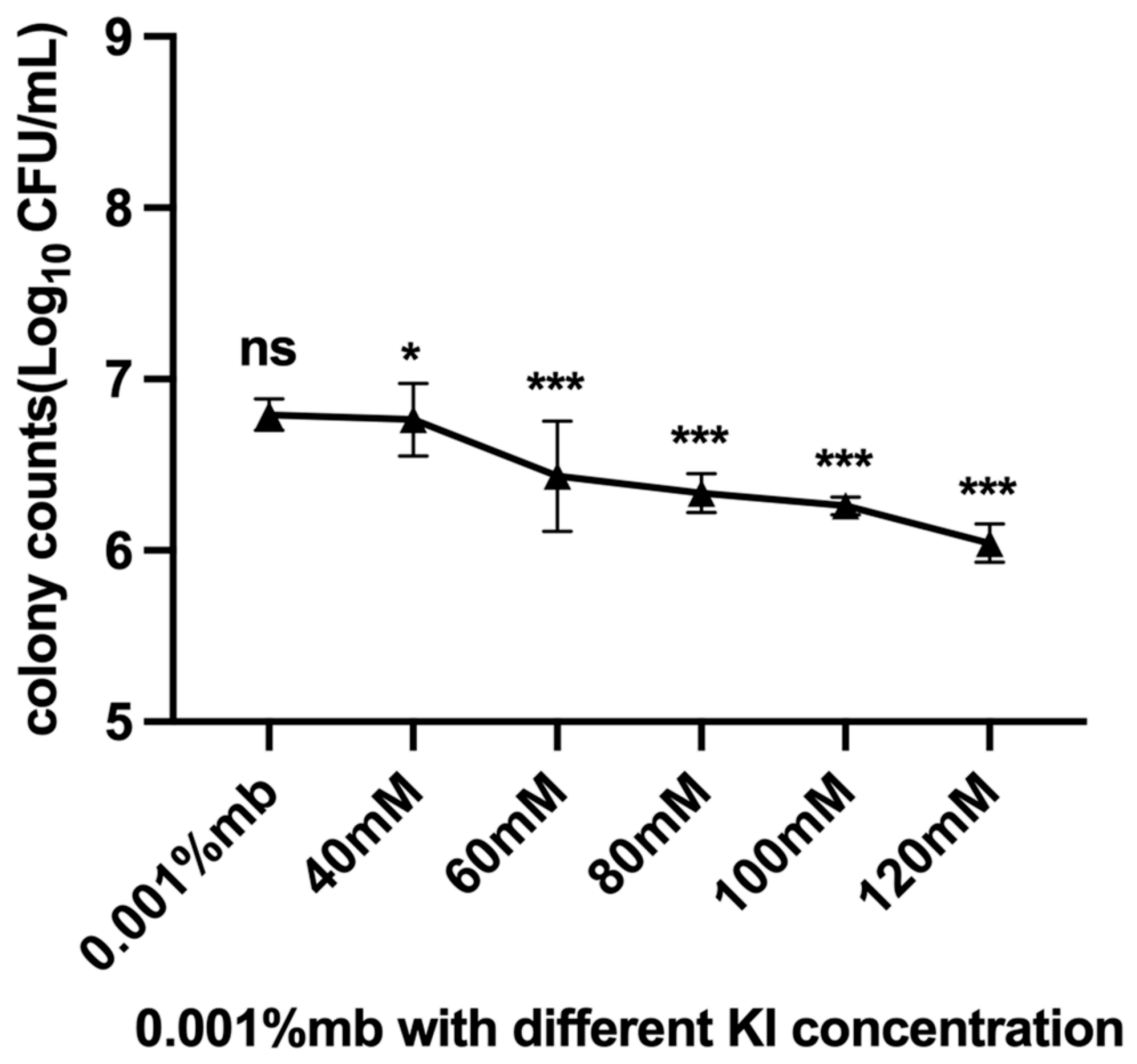
Screening of potassium iodide concentrations combined with MB-PDT. Viable bacterial counts after treatment with 0.001% MB-PDT alone or in combination with the indicated KI concentrations are expressed as Log10 CFU/mL. Data are presented as mean ± SD from six independent experiments (*n* = 6). Statistical comparisons were made with the 0.001% MB-PDT group without KI using one-way ANOVA followed by Tukey’s multiple-comparisons test. ns, not significant; * *p* < 0.05, *** *p* < 0.001.

Preliminary concentration-screening assays identified 0.001% MB and 80 mM KI as the lowest concentrations producing marked antibacterial effects; therefore, these concentrations were selected for the subsequent experiments (Figure 1 and Figure 2).

The antibacterial activity of MB-PDT, alone or in combination with KI, against cultured subgingival plaque-derived polymicrobial suspensions was evaluated using a colony-forming unit assay. The viable bacterial count in the untreated control group was 8.221 ± 0.140 Log10 CFU/mL. The viable bacterial counts in the light-only and KI-only groups did not differ significantly from that in the untreated control group (both *p* > 0.05), indicating no detectable independent antimicrobial effect under the tested conditions. Treatment with 0.001% MB-PDT significantly reduced the viable bacterial count to 6.793 ± 0.092 Log10 CFU/mL, corresponding to a 1.428-Log10 reduction relative to the untreated control group (*p* < 0.001).

**Figure 3:**
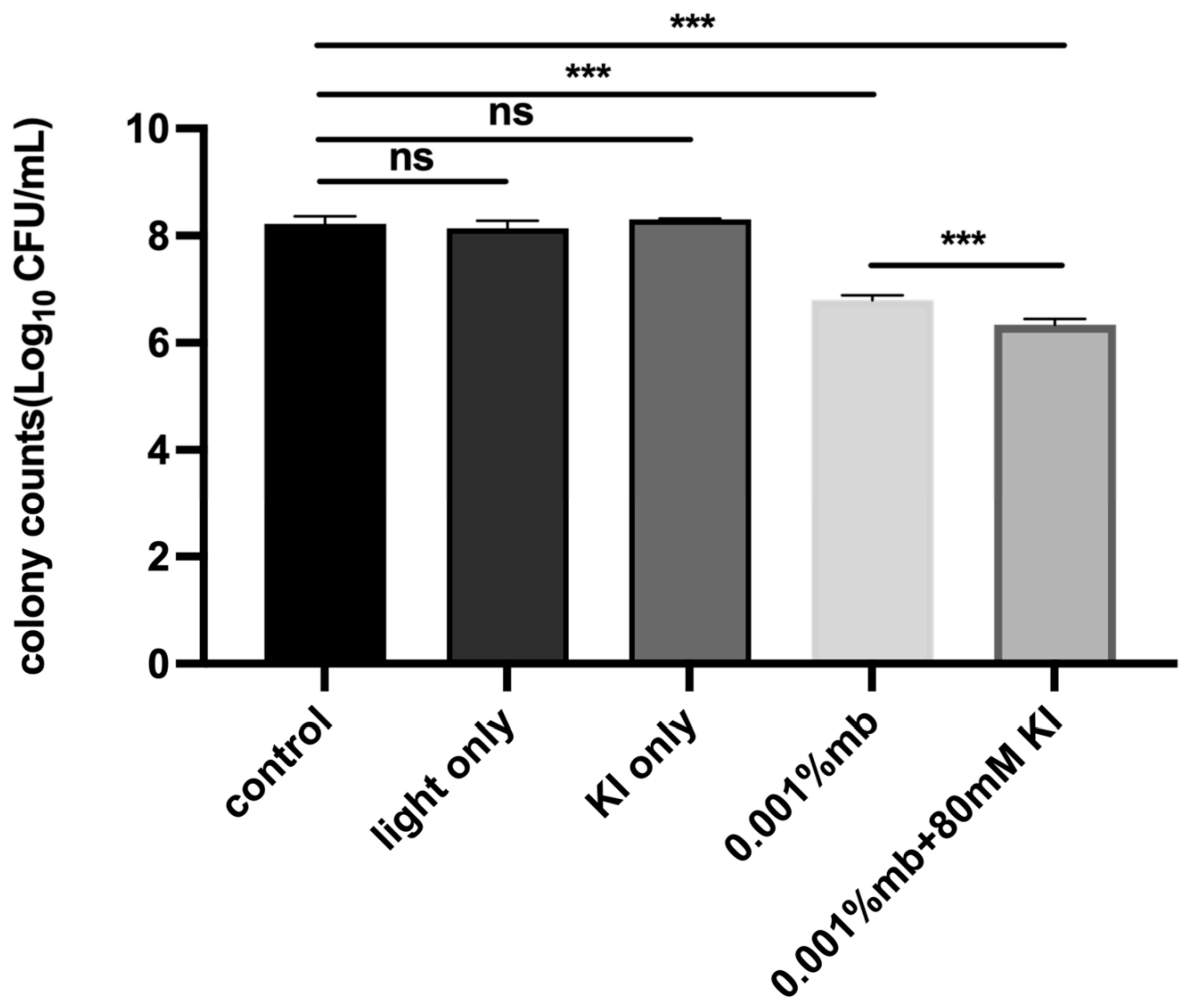
Antibacterial effects of control conditions and photodynamic treatments on cultured subgingival plaque-derived polymicrobial suspensions. The five groups were untreated control, light only, 80 mM KI only, 0.001% MB-PDT, and 0.001% MB plus 80 mM KI-PDT. Viable bacterial counts are expressed as Log10 CFU/mL. Data are presented as mean ± SD from six independent experiments (*n* = 6). Statistical comparisons were performed using one-way ANOVA followed by Tukey’s multiple-comparisons test; horizontal brackets indicate the corresponding pairwise comparisons. ns, not significant; *** *p* < 0.001.

The addition of 80 mM KI further reduced the viable bacterial count to 6.336 ± 0.114 Log10 CFU/mL, corresponding to a 1.885-Log10 reduction compared with the untreated control group (*p* < 0.001). The viable bacterial count in the MB + KI-PDT group was also significantly lower than that in the MB-PDT group, with an additional reduction of 0.457 Log10 CFU/mL (*p* < 0.001). These results demonstrate that KI enhanced the antibacterial activity of MB-PDT against the cultured subgingival plaque-derived polymicrobial suspension under the tested conditions (Figure 3).

### 3.2. LIVE/DEAD Staining of Cultured Subgingival Plaque-Derived Polymicrobial Suspensions After Different Treatments

**Figure 4:**
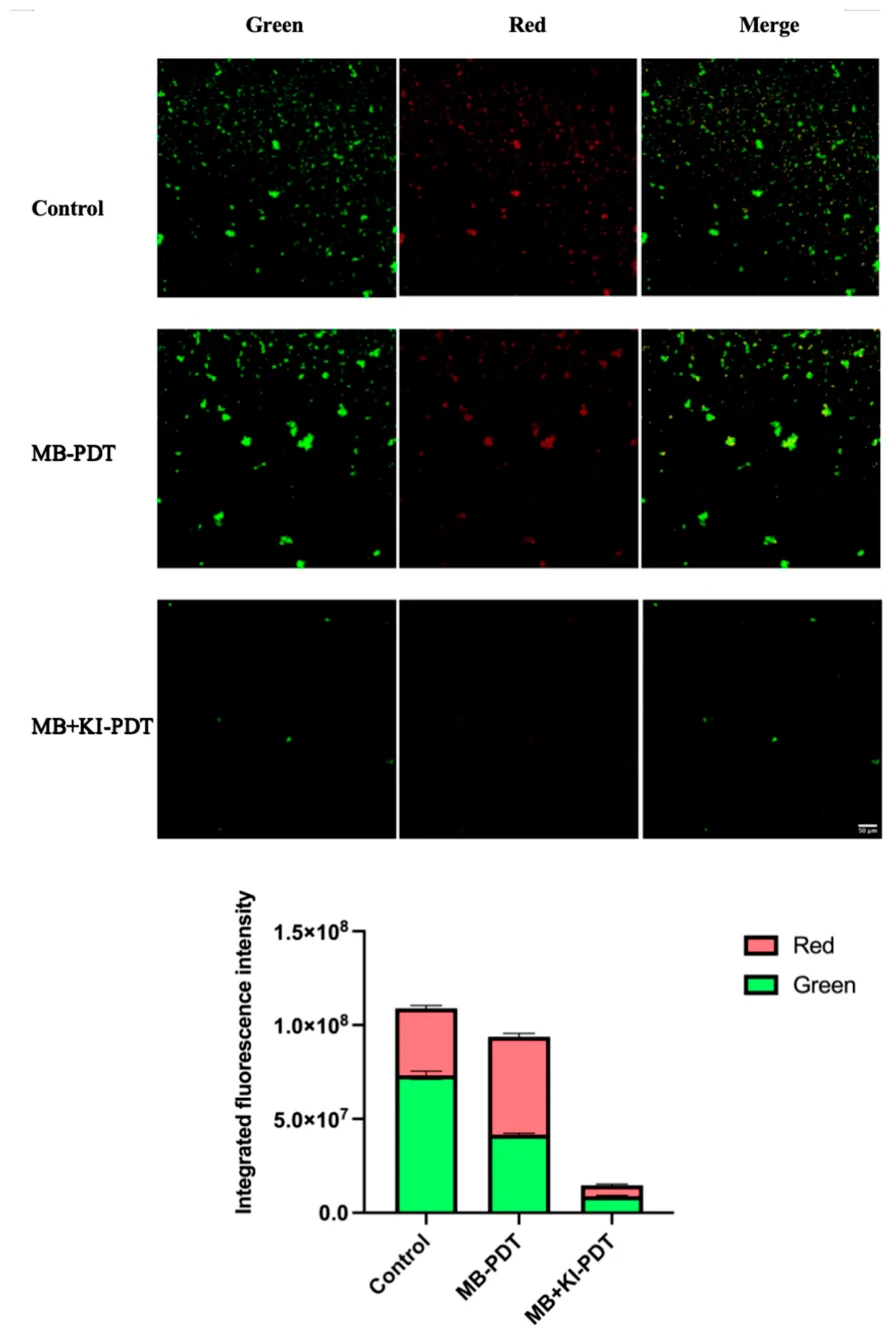
LIVE/DEAD staining and quantitative fluorescence analysis of cultured subgingival plaque-derived polymicrobial suspensions after the indicated treatments. Green SYTO 9 fluorescence represents bacteria with relatively intact membranes, whereas red propidium iodide fluorescence represents membrane-compromised bacteria. Integrated green and red fluorescence intensities were quantified using ImageJ and are shown in arbitrary units in the stacked bar graph. Quantitative data are presented as mean ± SD from six independent experiments (*n* = 6), and representative fluorescence images are shown. Scale bar = 50 μm.

As shown in Figure 4, the control group exhibited abundant green fluorescence and detectable red fluorescence. After MB-PDT treatment, the green fluorescence signal and the number of fluorescent bacterial cells decreased. The MB + KI-PDT group showed a further reduction in both green and red fluorescence signals. Only a few residual fluorescent bacterial cells were observed in the merged images of the MB + KI-PDT group. Quantitative fluorescence analysis further demonstrated a marked reduction in total fluorescence in the MB + KI-PDT group. The simultaneous reduction in green and red fluorescence may reflect extensive bacterial cell disruption, possible cell lysis, and the loss or degradation of stainable nucleic acids, leaving insufficient cellular material to retain either SYTO 9 or propidium iodide. Therefore, the weak red signal should not be interpreted as indicating bacterial survival.

### 3.3. MB-PDT Combined with KI Reduced the Expression of Virulence-Related Genes

**Figure 5:**
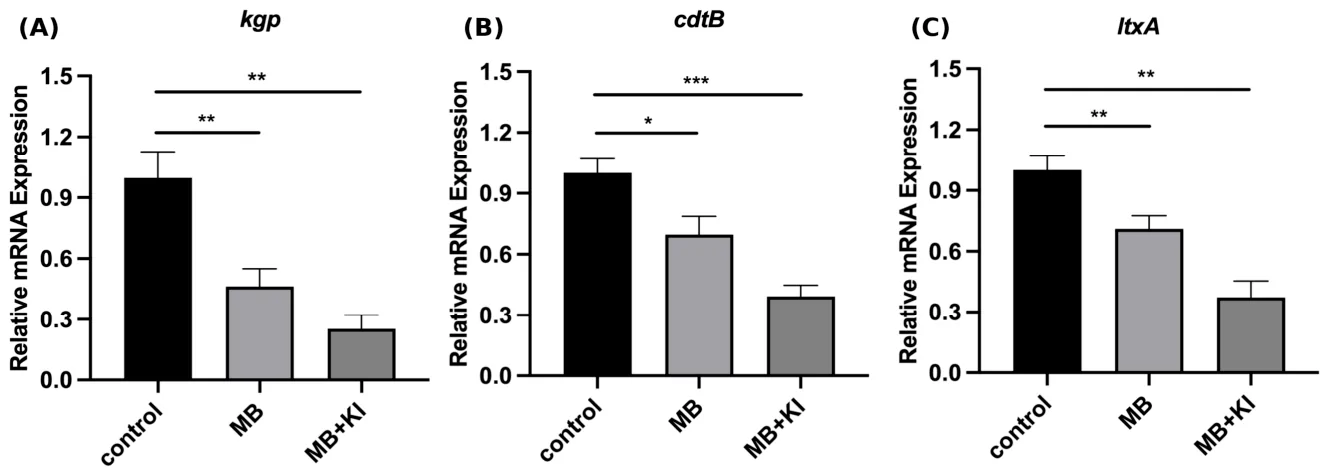
Relative expression of virulence-related genes after photodynamic treatment: (**A**) *kgp* in *Porphyromonas gingivalis*, (**B**) *cdtB* in *Fusobacterium nucleatum*, and (**C**) *ltxA* in *Aggregatibacter actinomycetemcomitans*. Expression levels were normalized to the corresponding species-specific 16S rRNA gene and calculated using the 2^−ΔΔCt method relative to the untreated control group. MB and MB + KI denote MB-PDT and MB + KI-PDT, respectively. Data are presented as mean ± SD from six independent experiments (*n* = 6). Statistical comparisons with the untreated control group were performed using one-way ANOVA followed by Tukey’s multiple-comparisons test. * *p* < 0.05, ** *p* < 0.01, *** *p* < 0.001.

The relative mRNA levels of virulence-related genes were detected by RT-qPCR. As shown in Figure 5A, the relative mRNA level of *kgp* in *P. gingivalis* was 0.997 ± 0.131 in the control group. After MB-PDT treatment, the relative mRNA level of *kgp* decreased to 0.463 ± 0.087, representing a 53.6% reduction compared with the control group (*p* < 0.01). In the MB + KI-PDT group, the relative mRNA level of kgp decreased to 0.253 ± 0.068, representing a 74.6% reduction compared with the control group (*p* < 0.01).

As shown in Figure 5B, for cdtB in *Fusobacterium nucleatum*, the relative mRNA level was 1.000 ± 0.070 in the control group. After MB-PDT treatment, the relative mRNA level of cdtB decreased to 0.697 ± 0.091, representing a 30.3% reduction compared with the control group (*p* < 0.05). In the MB + KI-PDT group, the relative mRNA level of cdtB decreased to 0.393 ± 0.055, representing a 60.7% reduction compared with the control group (*p* < 0.001).

As shown in Figure 5C, for ltxA in *Aggregatibacter actinomycetemcomitans*, the relative mRNA level was 1.000 ± 0.070 in the control group. After MB-PDT treatment, the relative mRNA level of ltxA decreased to 0.710 ± 0.066, representing a 29.0% reduction compared with the control group (*p* < 0.01). In the MB + KI-PDT group, the relative mRNA level of ltxA decreased to 0.373 ± 0.081, representing a 62.7% reduction compared with the control group (*p* < 0.01).

## 4. Discussion

Periodontal disease is an inflammatory condition triggered by plaque-derived microorganisms. In the present study, however, the clinical plaque samples were pooled, cultured on blood agar, harvested, and resuspended before treatment. Therefore, the experimental target was a cultured subgingival plaque-derived planktonic polymicrobial suspension rather than an intact subgingival biofilm. The results showed that MB-PDT reduced viable bacterial counts in this suspension model and that the addition of KI further enhanced the antimicrobial effect.

Photodynamic therapy (PDT) generates reactive oxygen species through interactions among photosensitizers, light, and molecular oxygen [19,20]. These reactive species damage microbial membranes, proteins, and nucleic acids. Methylene blue is widely used as a photosensitizer because of its water solubility and photochemical stability [12]. Previous studies have reported antimicrobial activity of MB-PDT against multidrug-resistant bacteria and in infected cutaneous squamous cell carcinoma [13]. In chronic periodontitis, adjunctive MB-PDT has been associated with reductions in pathogenic bacterial loads and improvements in clinical periodontal measures [21]. Consistent with these reports, MB-PDT reduced viable bacterial counts in the present cultured subgingival plaque-derived polymicrobial suspension. Because this model lacked native biofilm architecture, the result should not be extrapolated directly to intact clinical subgingival biofilms.

Although methylene blue-mediated photodynamic therapy (MB-PDT) exhibits inherent antibacterial properties, its efficacy as a standalone treatment can be constrained by factors including reactive oxygen species generation, treatment duration, and microbial tolerance. Consequently, strategies to augment the antibacterial performance of MB-PDT represent a key focus in contemporary research [22]. Potassium iodide (KI), an inorganic salt noted for its favorable water solubility and biocompatibility, can engage in oxidation processes during the photodynamic reaction, yielding iodine-based intermediates with antimicrobial activity that synergistically enhance the overall bactericidal outcome [23]. In the work of Li, Q. et al., KI supplementation was shown to promote the formation of additional free iodine (I_2_) and hydrogen peroxide (H_2_O_2_), thereby intensifying the lethal effect of MB-PDT against monospecies biofilms [16]. Similarly, Benine-Warlet J. et al. reported that KI potentiated the inactivation of Streptococcus mutans biofilms by red laser-activated methylene blue photodynamic therapy [24]. In the present study, the combined application of KI and MB-PDT yielded superior outcomes compared to MB-PDT alone, indicating that the role of KI extends beyond mere additive effects. Instead, KI likely amplifies microbial damage within the cultured subgingival plaque-derived polymicrobial suspension by actively participating in the oxidative cascade initiated by MB-PDT.

The study also examined relative transcript levels of three virulence-related genes. MB-PDT combined with KI was associated with lower normalized levels of *kgp*, *cdtB*, and *ltxA*. The *kgp* gene of *P. gingivalis* encodes a gingipain involved in tissue destruction, inflammatory regulation, and bacterial pathogenicity [25]. The *ltxA* gene of *Aggregatibacter actinomycetemcomitans* encodes a leukotoxin that can damage host leukocytes [26]. The *cdtB* gene detected in *Fusobacterium nucleatum* is associated with cytolethal distending toxin activity and host–cell DNA damage [27]. These results indicate lower species-normalized transcript levels after treatment; they do not by themselves establish reduced virulence in intact subgingival biofilms.

An important limitation of this study is that the experimental model did not retain the spatial organization or extracellular matrix of the native subgingival biofilm. Because biofilm architecture can restrict photosensitizer penetration and reduce the accessibility of reactive species, the antimicrobial efficacy observed in the present suspension model may not directly represent the response of an intact in situ biofilm. Although single melting peaks supported the specificity of the RT-qPCR primer pairs and corresponding species-specific 16S rRNA genes were used for within-species normalization, treatment-related changes in bacterial viability and community composition may still influence RNA-based measurements. Future studies should validate these findings using structured multispecies biofilm models, absolute species quantification, and clinical investigations.

In conclusion, potassium iodide enhanced the antimicrobial activity of methylene blue-mediated photodynamic therapy against cultured planktonic polymicrobial suspensions derived from clinical subgingival plaque. The combined treatment significantly reduced viable bacterial counts and was associated with lower relative transcript levels of the virulence-related genes *kgp*, *cdtB*, and *ltxA*. These findings are limited to the cultured subgingival plaque-derived polymicrobial suspension model and should not be interpreted as direct evidence of efficacy against intact subgingival biofilms.

## Figures and Tables

**Table 1:** Primer sequences used for RT-qPCR analysis.

Bacterial Species	Gene	GenBank Accession No.	Primer Sequence (5′–3′)	Amplicon Size (bp)
*Porphyromonas gingivalis*	16S rRNA	NR_118997.1	F: GGTGAGTAACGCGTAGGTAA R: ACGTCATCCCCACCTTCCT	141
*P. gingivalis*	kgp	CP003378.1 (PGN_1841); U09838.1	F: CGTCGGTATCGGTAACGAAG R: TTGCTGCTGTTGATGATGGC	120
*Fusobacterium nucleatum*	16S rRNA	NR_113664.1; CP003840.1	F: GTGAAATCTTGGAGATTTGCG R: CTTCCTCCGCTTATTGATATGC	194
*F. nucleatum*	cdtB	CP003840.1 (FN1793); AF109307.1	F: GCTGGTGCTGTTGTTGATGC R: TTGATGACCTTGCCGTTGAG	106
*Aggregatibacter* *actinomycetemcomitans*	16S rRNA	NR_118998.1; CP001071.1	F: GAGTTTGATCCTGGCTCAGAT TG R: AGGAGGTGATCCAGCCGCA	138
*A. actinomycetemcomitans*	ltxA	CP001071.1 (AAWG_00424); M29332.1	F: ATGCGGCTTTTCTACCGTCT R: CGTCGATGATCTTGGTGCTG	115

**Note:** 16S rRNA was used as the internal reference gene for the corresponding bacterial species. F, forward primer; R, reverse primer.

## Data Availability

The data presented in this study are available from the corresponding author upon reasonable request.

## References

[1] Niemiec B.A. (2008). Periodontal disease. Top. Companion Anim. Med..

[2] Kinane D.F. (2001). Causation and pathogenesis of periodontal disease. Periodontology 2000.

[3] Kwon T., Lamster I.B., Levin L. (2021). Current Concepts in the Management of Periodontitis. Int. Dent. J..

[4] Valm A.M. (2019). The Structure of Dental Plaque Microbial Communities in the Transition from Health to Dental Caries and Periodontal Disease. J. Mol. Biol..

[5] Ding J., Zhao C., Gao L. (2023). Metabolism of periodontal pathobionts: Their regulatory roles in the dysbiotic microbiota. Mol. Oral Microbiol..

[6] Sanz M., Herrera D., Kebschull M., Chapple I., Jepsen S., Berglundh T., Sculean A., Tonetti M.S., EFP Workshop Participants and Methodological Consultants (2020). Treatment of stage I-III periodontitis-The EFP S3 level clinical practice guideline. J. Clin. Periodontol..

[7] Cobb C.M., Sottosanti J.S. (2021). A re-evaluation of scaling and root planing. J. Periodontol..

[8] Cieplik F., Deng D., Crielaard W., Buchalla W., Hellwig E., Al-Ahmad A., Maisch T. (2018). Antimicrobial photodynamic therapy—What we know and what we don’t. Crit. Rev. Microbiol..

[9] Alves E., Faustino M.A., Neves M.G., Cunha A., Tome J., Almeida A. (2014). An insight on bacterial cellular targets of photodynamic inactivation. Future Med. Chem..

[10] Sun Y., Ogawa R., Xiao B.H., Feng Y., Wu Y., Chen L., Gao X., Chen H. (2020). Antimicrobial photodynamic therapy in skin wound healing: A systematic review of animal studies. Int. Wound J..

[11] Wainwright M. (2005). The development of phenothiazinium photosensitisers. Photodiagn. Photodyn. Ther..

[12] Tardivo J.P., Del Giglio A., de Oliveira C.S., Gabrielli D.S., Junqueira H.C., Tada D.B., Severino D., Turchiello R.d.F., Baptista M.S. (2005). Methylene blue in photodynamic therapy: From basic mechanisms to clinical applications. Photodiagn. Photodyn. Ther..

[13] Xu L., Li X., Zhao C., Zhang X., Kou H., Yang Y., Zhu F., Zhang W., Lu Y. (2021). A combination of preoperative or intraoperative MB-PDT and surgery in the treatment of giant cutaneous squamous cell carcinoma with infection. Photodiagn. Photodyn. Ther..

[14] Hamblin M.R. (2017). Potentiation of antimicrobial photodynamic inactivation by inorganic salts. Expert. Rev. Anti Infect. Ther..

[15] Yuan L., Lyu P., Huang Y.Y., Du N., Qi W., Hamblin M.R., Wang Y. (2020). Potassium iodide enhances the photobactericidal effect of methylene blue on Enterococcus faecalis as planktonic cells and as biofilm infection in teeth. J. Photochem. Photobiol. B..

[16] Li Q., Liu Y., Zheng J., Chen Y., Liu Z., Xie Q., Li D., Xi L., Zheng J., Liu H. (2024). Potassium iodide enhances the killing effect of methylene blue mediated photodynamic therapy against *F. monophora*. Photodiagn. Photodyn. Ther..

[17] Papapanou P.N., Sanz M., Buduneli N., Dietrich T., Feres M., Fine D.H., Flemmig T.F., Garcia R., Giannobile W.V., Graziani F. (2018). Periodontitis: Consensus report of workgroup 2 of the 2017 World Workshop on the Classification of Periodontal and Peri-Implant Diseases and Conditions. J. Clin. Periodontol..

[18] Hajishengallis G., Lamont R.J. (2012). Beyond the red complex and into more complexity: The polymicrobial synergy and dysbiosis (PSD) model of periodontal disease etiology. Mol. Oral Microbiol..

[19] Carrera E.T., Dias H.B., Corbi S.C.T., Marcantonio R.A.C., A Bernardi A.C., Bagnato V.S., Hamblin M.R., Rastelli A.N.S. (2016). The application of antimicrobial photodynamic therapy (aPDT) in dentistry: A critical review. Laser Phys..

[20] Singh S., Nagpal R., Manuja N., Tyagi S.P. (2015). Photodynamic therapy: An adjunct to conventional root canal disinfection strategies. Aust. Endod. J..

[21] Perumal M.K.K., Renuka R.R., Natarajan P.M. (2024). Evaluating the potency of laser-activated antimicrobial photodynamic therapy utilizing methylene blue as a treatment approach for chronic periodontitis. Front. Oral Health.

[22] Cecatto R.B., de Magalhães L.S., Rodrigues M.F.S.D., Pavani C., Lino-Dos-Santos-Franco A., Gomes M.T., Silva D.F.T. (2020). Methylene blue mediated antimicrobial photodynamic therapy in clinical human studies: The state of the art. Photodiagn. Photodyn. Ther..

[23] Vecchio D., Gupta A., Huang L., Landi G., Avci P., Rodas A., Hamblin M.R. (2015). Bacterial photodynamic inactivation mediated by methylene blue and red light is enhanced by synergistic effect of potassium iodide. Antimicrob. Agents Chemother..

[24] Benine-Warlet J., Brenes-Alvarado A., Steiner-Oliveira C. (2022). Potassium iodide enhances inactivation of Streptococcus mutans biofilm in antimicrobial photodynamic therapy with red laser. Photodiagn. Photodyn. Ther..

[25] Curtis M.A., Aduse Opoku J., Rangarajan M., Gallagher A., Sterne J.A.C., Reid C.R., Evans H.E.A., Samuelsson B. (2002). Attenuation of the virulence of Porphyromonas gingivalis by using a specific synthetic Kgp protease inhibitor. Infect. Immun..

[26] Vega B.A., Belinka B.A., Kachlany S.C. (2019). *Aggregatibacter actinomycetemcomitans* Leukotoxin (LtxA; Leukothera^®^): Mechanisms of Action and Therapeutic Applications. Toxins.

[27] Yamano R., Ohara M., Nishikubo S., Fujiwara T., Kawamoto T., Ueno Y., Komatsuzawa H., Okuda K., Kurihara H., Suginaka H. (2003). Prevalence of cytolethal distending toxin production in periodontopathogenic bacteria. J. Clin. Microbiol..

